# Statistical Analysis of Zebrafish Locomotor Response

**DOI:** 10.1371/journal.pone.0139521

**Published:** 2015-10-05

**Authors:** Yiwen Liu, Robert Carmer, Gaonan Zhang, Prahatha Venkatraman, Skye Ashton Brown, Chi-Pui Pang, Mingzhi Zhang, Ping Ma, Yuk Fai Leung

**Affiliations:** 1 Department of Statistics, University of Georgia, Athens, Georgia, United States of America; 2 Department of Biological Sciences, Purdue University, West Lafayette, Indiana, United States of America; 3 Department of Statistics, Purdue University, West Lafayette, Indiana, United States of America; 4 Department of Ophthalmology and Visual Sciences, Chinese University of Hong Kong, Hong Kong, China; 5 Joint Shantou International Eye Center, Shantou University & the Chinese University of Hong Kong, Shantou, China; 6 Department of Biochemistry and Molecular Biology, Indiana University School of Medicine Lafayette, West Lafayette, Indiana, United States of America; National Institutes of Health / NICHD, UNITED STATES

## Abstract

Zebrafish larvae display rich locomotor behaviour upon external stimulation. The movement can be simultaneously tracked from many larvae arranged in multi-well plates. The resulting time-series locomotor data have been used to reveal new insights into neurobiology and pharmacology. However, the data are of large scale, and the corresponding locomotor behavior is affected by multiple factors. These issues pose a statistical challenge for comparing larval activities. To address this gap, this study has analyzed a visually-driven locomotor behaviour named the visual motor response (VMR) by the Hotelling’s T-squared test. This test is congruent with comparing locomotor profiles from a time period. Different wild-type (WT) strains were compared using the test, which shows that they responded differently to light change at different developmental stages. The performance of this test was evaluated by a power analysis, which shows that the test was sensitive for detecting differences between experimental groups with sample numbers that were commonly used in various studies. In addition, this study investigated the effects of various factors that might affect the VMR by multivariate analysis of variance (MANOVA). The results indicate that the larval activity was generally affected by stage, light stimulus, their interaction, and location in the plate. Nonetheless, different factors affected larval activity differently over time, as indicated by a dynamical analysis of the activity at each second. Intriguingly, this analysis also shows that biological and technical repeats had negligible effect on larval activity. This finding is consistent with that from the Hotelling’s T-squared test, and suggests that experimental repeats can be combined to enhance statistical power. Together, these investigations have established a statistical framework for analyzing VMR data, a framework that should be generally applicable to other locomotor data with similar structure.

## Introduction

Zebrafish have revolutionized high-throughput neurobehaviour research. One key reason is that zebrafish routinely produce a large number of embryos [1]. These embryos are small and can be arrayed in multi-well culture plates. This systematic arrangement makes it simple to simultaneously measure locomotor behaviour of many embryos under external simulation. Indeed, the resulting data have provided new insights into neurobiology [2–5], pharmacology [4,6] and toxicology [7–11]. Nonetheless, the complexity of these locomotor data has created new challenges in data analysis, which have not been thoroughly explored. This has potentially limited what can be learned from the high-throughput neurobehaviour studies.

The analytical challenges of these studies originate from the experimental setup, data collection and data structure. To illustrate these challenges, we will describe a popular high-throughput analysis of larval fish behaviour: the visual motor response (VMR). This response is a locomotor behaviour stimulated by drastic light onset or offset [5,12–14]. In a typical VMR experiment, zebrafish larvae are arranged in a 96-well plate, isolated from environmental light in a lightproof chamber, and stimulated by controlled white light. Using this setup, the movement of multiple larvae can be simultaneously measured. For example, these larvae are detected as pixels in the video. When the detected pixels exceed a pre-defined threshold in successive frames of the video, the larvae are deemed moving [4,5,11–15]. Another approach to detect larval movement is to track the larval displacement between successive frames [8–10,16–20]. In some studies, the displacement will also be transformed into velocity. All these activity measures are quantified over a period of time to generate time-series activity data for downstream analysis.

The first step of analysis is to visualize the collected data in a plot. Typically, the activity values are averaged across the same type of sample, and plotted against time to illustrate the general behavioral profile of the animals [5,14]. In this case, the resulting locomotor activity is often reported as activity value per unit time. Alternatively, the larval activity can be visualized by a heatmap [21], or by mapping the results back to individual wells of the multi-well plate [22]. These data plots can qualitatively compare activity between different types of sample [5,12,14,15]. For quantitative comparison, the locomotor data are often analyzed by standard statistical tests including t-test and analysis of variance (ANOVA) [8,10,11,16,18]. The dynamical change of behaviour has also been analyzed with repeated-measures ANOVA [9,17].

However, these statistical tests may not be the most suitable for analyzing VMR data. For example, t-test can only deal with one factor with two levels at a time. Its overall Type I error rate will increase with the number of simultaneous tests on multiple factors with multiple levels. Moreover, t-test does not take into account of time dependence, a situation in which the data obtained from samples repeatedly measured at different times are correlated in time. This time dependence feature is inherent in behavioural data, and is better handled by repeated-measures ANOVA. This test is primarily used when subjects are repeatedly measured or experimented under different conditions, and it extracts between-subjects variations from the error variance. However, repeated-measures ANOVA requires data variance satisfying sphericity assumption, an assumption that stipulates the variances of the differences between all possible pairs of groups are equal. This assumption is hardly satisfied in behavioural studies because of drastically different variances under distinct conditions. To handle the lack of appropriate statistical tests, several studies have transformed the activity values into a format that is amenable to simple parametric analysis. For example, they can be transformed into total displacement, mean velocity, or swimming time of each larva. These transformed values can then be used in standard parametric analyses [8,9,16,17]. This approach is useful for detecting differences in the transformed activity values in a specific time period. Using this approach, Lange and colleagues found that different WT strains swam different distances, in different speed, and for different time in an hour [16]. Nonetheless, data transformation unavoidably discards information from the original data, and may result in a loss in sensitivity for statistical analysis. In addition to this limitation, there are also inherent experimental and biological variations of the VMR setup that have not been formally studied. These include variation between biological replicates, and variation among wells in the assay due to the potential unevenness of light illumination. These statistical issues are limiting the utility of VMR in various behavioral investigations.

To address these statistical challenges, we have established a coherent statistical analysis framework for VMR data analysis. The framework was developed using a dataset collected from three wild-type (WT) zebrafish strains: AB, TL and TLAB, from 3 to 9 days postfertilization (dpf). To compare activities between different conditions, we applied the Hotelling’s T-squared test. This test is the generalization of the conventional t-test, which tends to result in an inflated error rate due to multiple testing on data like the situations outlined in this study. In contrast, Hotelling’s T-squared test gives an overall test and reduces the corresponding error rate. As a result, it performs better than the t-test. This study illustrates the utility of the Hotelling’s T-squared test by comparing the VMR data in several commonly-used scenarios, and by conducting a power analysis that indicates the number of samples required is compatible with existing study designs. Furthermore, factors that might affect larval activity were evaluated by multivariate analysis of variance (MANOVA) models. MANOVA does not require the input data to satisfy sphericity assumption, and is more suitable for VMR dataset than repeated-measures ANOVA. Together, this study has established an analysis framework using VMR data that share similar data structure with other locomotor data. Thus, this analysis framework should be generally applicable to similar locomotor data collected in various neurobehaviour studies.

## Materials and Methods

### 2.1 Experimental Design and Data Collection

#### 2.1.1. Zebrafish maintenance, breeding and embryo collection

In this study, the following WT zebrafish strains were used: AB, TL and TLAB (AB/TL; A hybrid of AB and TL) <http://zfin.org/action/feature/wildtype-list?MIval=aa-wtlist.apg>. They were maintained and bred in groups of two females and two to four males according to standard procedures [23]. The collected embryos were maintained in E3 medium at 28°C in an incubator with the same light-dark cycle as in the fish facility. The medium was changed every day and unhealthy embryos were discarded. At 3 dpf, healthy embryos were further selected based on the following criteria: no visible physical defects such as bent spines, bloated bodies or other deformities. These healthy embryos were transferred to a 96-well plate for use in the subsequent behavioural assay. All these protocols were approved by the Purdue Animal Care and Use Committee.

#### 2.1.2. The visual motor response (VMR) Assay

**Fig 1:**
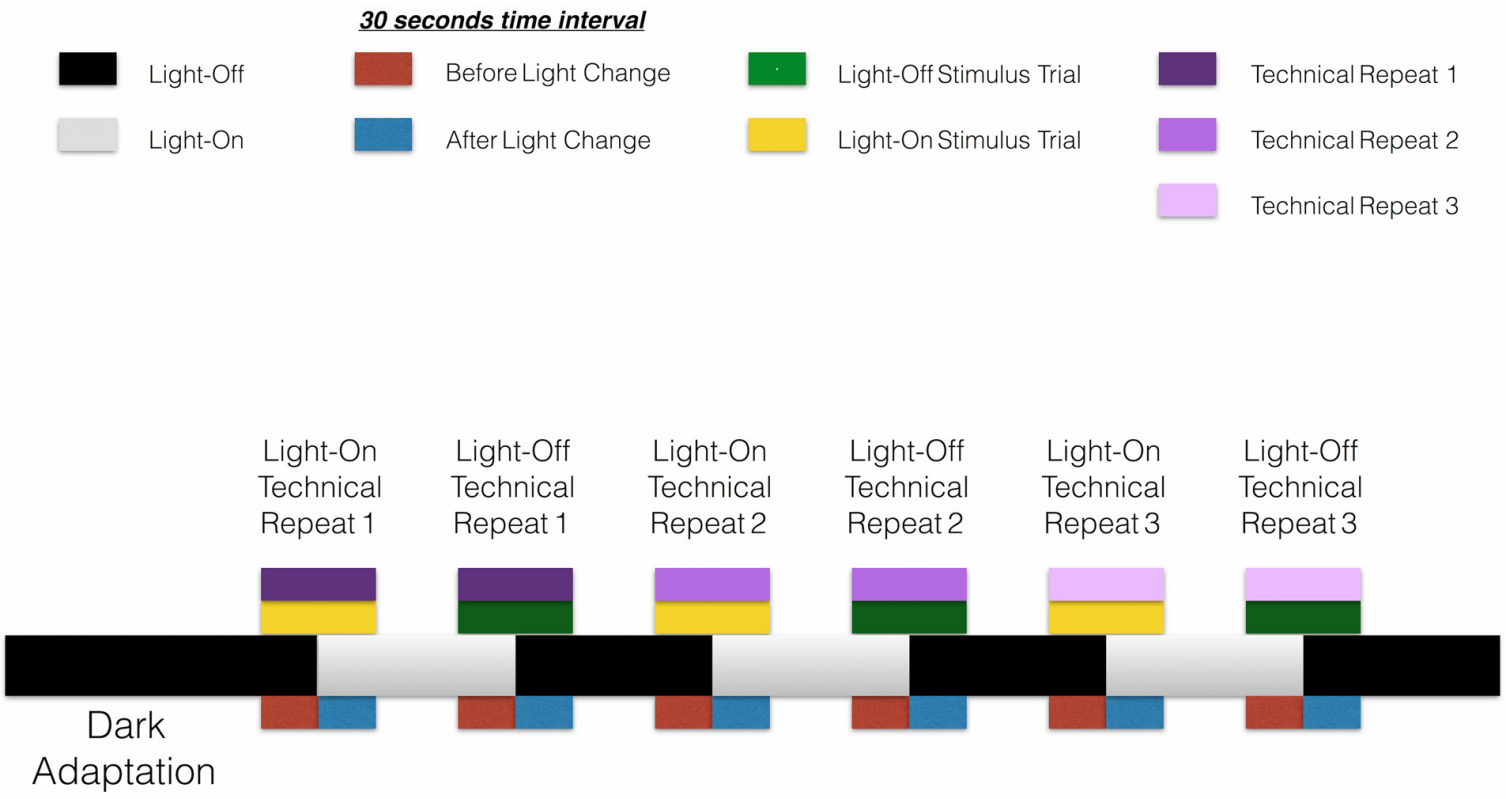
VMR experimental scheme. The experimental scheme for the VMR assay was adopted from Emran and colleague (2008). In the scheme, the larvae arrayed in the 96-well plate were first dark adapted for 3.5 hrs (long black bar on the left). Then, they were subjected to three consecutive trials of light onset (Light-On; grey bars) and light offset (Light-Off; short black bars). Each Light-On or Light-Off session lasted for 30 mins. In this study, we extracted the data from 30 s before light change (red bars; not to scale) to 30 s after light change (blue bars; not to scale) for statistical analyses. In some cases, the analyses separately handled the extracted data around the Light-On stimulus (yellow boxes) and Light-Off stimulus (green boxes). Furthermore, in our MANOVA models, the effect of three consecutive trials was explicitly evaluated (purple boxes in different colour values), regardless of the nature of light change.

The VMR assay was implemented based on the design by Emran and colleagues [5,12] as described below. The assay was conducted inside the ZebraBox system (ViewPoint Life Sciences, Lyon, France). In the system, the 96-well plate with the animals was isolated from environmental light, and stimulated by white light emitted by a light-controlling unit from the bottom of the plate. The animal movement was recorded by an infra-red camera at a rate of 30 frames per second under infra-red light illumination at 850 nm, which the animals could not perceive. Before the actual experiment, the 96-well plate with the animals was placed in the ZebraBox system for 3.5 hours of dark adaption to acclimatize the animals. The data collection was started at 0.5 hours before the first light onset. The actual test consisted of three consecutive trials of light onset (Light-On) and light offset (Light-Off) periods with each period lasted for 30 minutes (Fig 1). The light change (On or Off) was abrupt and was not fading. The Light-On stimulus was set at 100% of the output intensity, which was measured by a LX1010B light meter (Mastech, Taipei, Taiwan). The measurements were taken at nine evenly distributed locations across the surface of the light-controlling unit that would be covered by the 96-well plate. The mean illuminance of these nine locations was 1390.94 Lux and the standard deviation of the measurement was 155.05 Lux. Using this experimental scheme, the VMR was measured from the three WT strains. For each strain, two biological repeats were conducted. Each repeat was started with 96 embryos in the 96-well plate at 3 dpf. The VMR of the same animals in each biological repeat was measured from 3 to 9 dpf. In each day, the assay was started at around the same time at 2 p.m. The health of the animals was also inspected every day. During the inspection, half of the E3 medium was changed. Embryos or larvae that showed any signs of abnormality during the course of behavioural experiment were excluded from the final data analysis. The remaining healthy embryos were not fed during the experimental period to avoid any confounding errors introduced by feeding. All data are available at the Harvard Dataverse (http://dx.doi.org/10.7910/DVN/HTXXKW).

### 2.2. Statistical Analysis

#### 2.2.1. Activity summarization

To detect movement from the video data, the ZebraBox machine used the following method: First, each embryo was first detected in each frame by registering pixels with a grey level below a preset level. This level was defined as the detection sensitivity and was set at 6 in this study. If the registered pixels were different in successive frames, they were declared as active pixels. These active pixels represented the part of the animal that moved in successive frames. Small movements could be separated from major moving episodes by setting a burst threshold to select movements that were larger than a predefined number of active pixels between successive frames. This burst-threshold filter was not used in this study because we recently showed that small movements could provide useful information for better identification of animals with different genotype [13]. Then, the larval movement was summarized as the fraction of frames in each second with active pixels/movement, a parameter that was defined as the Burst Duration. The data in each second summarized the activities from the second before to that particular second. For example, data collected in second 1 summarized all activities between second 0 (when light change occurred) and second 1. Finally, the Burst Duration was computed for each animal at every second of the assay to measure the activity level of the animal.

#### 2.2.2. Data modeling and statistical inference

Upon abrupt light change, the activity level of the animals can reveal underlying visual problem and can potentially be used to identify new drugs [5,13–15]. These properties are of high interest to our research; thus, we extracted the activity data from 30 seconds before light change (Fig 1, red boxes) to 30 seconds after light change (Fig 1, blue boxes) to conduct statistical analysis. During this transitional period, the larval activity was influenced by many factors, which were incorporated in our models. These factors will be defined and grouped by their nature in section 2.2.2.1. Then, the effect of these factors on larval activity will be studied by the Hotelling’s T-squared test and MANOVA model in sections 2.2.2.2 and 2.2.2.3 respectively. All statistical analyses were performed with R software version 3.2.0 <http://www.r-project.org/>. The analysis scripts are available in S1 File.

2.2.2.1. Model parameters:

1. Biological variations
  - Strains. Three WT strains were used in the experiment: AB, TL and TLAB.
  - Stages. The stage of zebrafish ranged from 3 to 9 dpf.
2. Treatment effects
  - Technical Repeat. The VMR experiment design has three technical repeats: three light onsets (Light-On), and three light offsets (Light-Off). This factor studies the effects of repeated light change regardless of light onset or offset (Fig 1, purple boxes in three different colour values).
  - Light Stimulus. This factor separates the change in activity caused by the Light-On sessions (Fig 1, yellow boxes) and Light-Off sessions (Fig 1, green boxes).
3. Experimental variations
  - Location. The VMR assay was conducted with fish larvae arranged in a 96-well plate. The difference in their physical location inside the plate may contribute to variations in activity.
  - Biological Repeat. For each WT strain, there were two independent replications conducted with embryos collected on different days.
4. Interactions between the factors under categories 1–3 above.

***2*.*2*.*2*.*2*. *Hotelling’s T-squared test***: The two-sample Hotelling’s T-squared test is the multivariate version of the two-sample t-test in univariate statistics. This test enables the comparison between mean vectors. Compared to t-test, it maintains the data structure and takes into account of the correlation between measured traits. In univariate statistics, the t-statistic is usually defined as the difference between sample mean $\bar{x}$ and population mean *μ*,

$$t\;=\sqrt{n}\;\frac{\bar{x}-\mu }{s},$$

when the sample comes from a normal distribution *N*(*μ*, *σ* ^2^), and *n* is the sample size, *s* is sample variance. The multivariate analog of the squared t in univariate case is given by

$$T^{2}=\frac{n_{1}n_{2}}{n_{1}+n_{2}}{\left({\bar{X}}^{\left(1\right)}-{\bar{X}}^{\left(2\right)}\right)}^{\prime }S^{-1}\left({\bar{X}}^{\left(1\right)}-{\bar{X}}^{\left(2\right)}\right),$$

where ${\bar{X}}^{\left(1\right)}$ and ${\bar{X}}^{\left(2\right)}$ with length *p*, are the mean vectors of a sample of size *n*_1_ and *n*_2_ respectively, and S is the weighted sample covariance matrix. Under the null hypothesis, the test statistics $\frac{n_{1}+n_{2}-p-1}{p\left(n_{1}+n_{2}-2\right)}\;T^{2}$ follows F-distribution with degrees of freedom *p* and *n*_1_ + *n*_2_ − *p* − 1. To control the probability of committing type I error in simultaneous comparisons of Hotelling’s T-squared test, p-values of these tests are adjusted by controlling the false discovery rate [24].

Further, to obtain the power of Hotelling’s T-squared test [25], we need the following power function:

$$P\left(F_{p,\left(n_{1}+n_{2}-p-1\right),\tau^{2}}\geq F_{p,\left(n_{1}+n_{2}-p-1\right),\;\alpha }\right),$$

where $F_{p,\left(n_{1}+n_{2}-p-1\right),\tau^{2}}$ is the non-central F-distribution with noncentrality parameter *τ* ^2^, and *p* and *n*_1_ + *n*_2_ − *p* − 1 are the degrees of freedom. $F_{p,\left(n_{1}+n_{2}-p-1\right),\;\alpha }$ denotes the density value of central F-distribution with degrees of freedom *p* and *n*_1_ + *n*_2_ − *p* − 1 given the significance level *α*. Denote that *i* ^*th*^(*i* = 1,2) group of samples $X_{1}^{\left(i\right)},\;\ldots ,\;X_{n_{i}}^{\left(i\right)}$ comes from *N*(*μ* ^(*i*)^, *Σ*), for the two-sample test of H_0_:*μ* ^(1)^ = *μ* ^(2)^, the noncentrality parameter is calculated as

$$\tau^{2}=\;\frac{n_{1}n_{2}}{n_{1}+n_{2}}{\left(\mu^{\left(1\right)}-\mu^{\left(2\right)}\right)}^{\prime }\Sigma^{-1}\left(\mu^{\left(1\right)}-\mu^{\left(2\right)}\right).$$

Given the difference *δ* = *μ* ^(1)^ − *μ* ^(2)^ and the significance level *α*, we may choose *n*_1_ and *n*_2_ so that *τ* ^2^ is sufficiently large to be able to reject the null hypothesis. Given the power under a certain significance level, we can also calculate the noncentrality parameter *τ* ^2^, which indicates the amount of difference that a test could detect. Using this framework, a simulation study was conducted to study the relationship between statistical power, sample size, and activity profile difference that the Hotelling’s T-squared test could detect. In the simulation, *μ* ^(1)^ and *μ* ^(2)^ were generated from two different uniform distributions, and the population variance-covariance matrix was estimated by sample variance-covariance matrix calculated from the data. Then, the power of the test was calculated across different sample sizes ranged from 16 to 100 each group given that *α* = 0.05. The simulation results were plotted in power curves to visualize the relationship between the power of Hotelling’s T-squared test and the sample size of each group. Furthermore, we conducted a power analysis using the activity values of two WT stains: AB and TLAB at 6 dpf to illustrate the performance of the test on real data.

***2*.*2*.*2*.*3*. *MANOVA model***: Different factors might contribute differently to the final activity of the zebrafish larvae. Therefore, we developed a multi-factor MANOVA model to compare the effects of those factors under different conditions. MANOVA is the multivariate analogue of conventional ANOVA, and has been widely used to compare multivariate response of multiple groups. In MANOVA, we calculated the hypothesis sum of squares and cross product (SSCP), and the error SSCP. The two SSCP terms are similar to the sum of squares in conventional ANOVA. When testing significance using F-test, we used Pillai-Bartlett Trace based on the SSCP [25]. Pillai-Bartlett Trace is similar to the sum of variance that is explained by the factors in model. It was used to indicate the effect size of each factor in the MANOVA model.

To study the dynamic effect of each factor over time, we also performed univariate ANOVA for each second, using the dependent variables in the MANOVA model. The association between main effects and dependent variable was measured by the effect size *η* ^2^, which is the proportion of variance in the dependent variable that is attributed to each effect. It is defined as

$$\eta^{2}=\frac{SS_{effect}}{SS_{total}}\;,$$

where SS represents the sums of squares, *SS*_*effect*_ is the sums of squares for effect, and *SS*_*total*_ is the total sums of squares.

## Results

**Fig 2:**
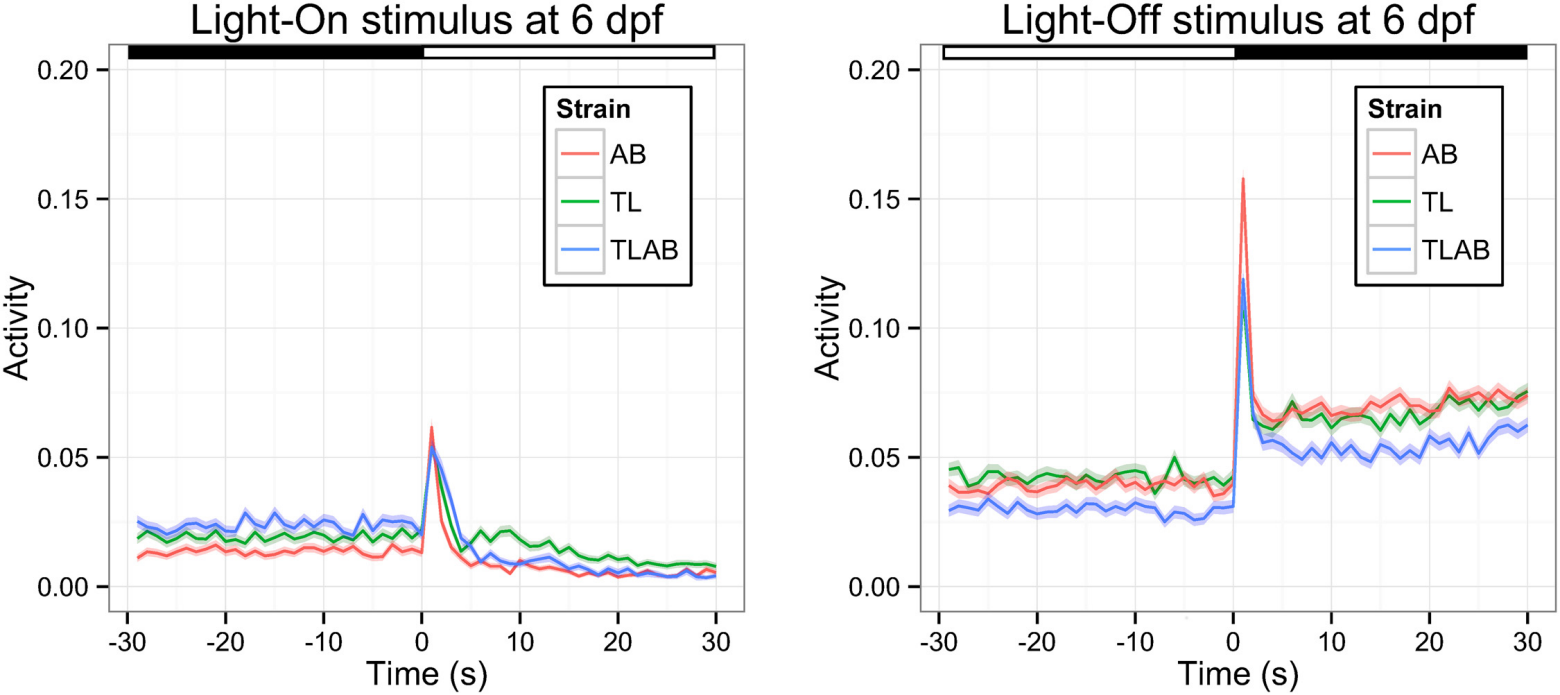
The VMR of 6-dpf WT strains. The VMR was measured from three WT strains: AB, TL and TLAB as described in the methods. The activity was defined as the fraction of frames in each second that a larva in the video was detected moving (see methods for the detailed definition). Then, the activities from all trials under the same light stimulus were averaged and plotted. The results for Light-On and Light-Off VMR are shown in the left and right figures respectively. These figures show the mean activity of AB (red trace), TL (green trace) and TLAB (blue trace) from 30 s before the light change to 30 s after the light change. The corresponding error in 1 S.D. is shown by the colour ribbon surrounding the mean activity trace. The light and dark periods are indicated by white and black bars at the top of the figures. The sample size was 573 for AB, 528 for TL, and 555 for TLAB respectively. These values are the total observation from the two biological repeats and three technical repeats.

We measured the VMR of the larvae obtained from three WT strains: AB, TL, and TLAB. These larvae showed distinct behaviors, as indicated by the mean larval activity of each strain during light onset (Light-On) and light offset (Light-Off). An example of this difference at 6 dpf is shown in Fig 2.

### 3.1. Hotelling’s T-squared test

The plots in Fig 2 qualitatively show the difference between the activity of different strains, and between the activity before and after light change. However, these plots do not provide a quantitative measurement of the observed differences. To this end, a two-sample Hotelling’s T-squared test was used for pairwise comparison of selected conditions. This approach is illustrated by the following four examples:

#### 3.1.1. Example 1: Difference between strains during the same time interval

To determine the VMR difference between WT strains as shown in Fig 2, a pairwise Hotelling’s T-squared test was performed on the WT activity data across different strains during these two time periods: -29–0 s (i.e., before light change) and 1–30 s (i.e., after light change) (Table 1A). The Light-On and Light-Off stimuli were separately analyzed. For the 30-s period before Light-On stimulus (-29–0 s), there was no statistical difference in the activity between AB & TL (p = 0.1017), and TL & TLAB (p = 0.2572), while there was a difference in the activity between AB & TLAB (p < 0.0001). These are supported by the activity plots from -29–0 s (Fig 2, left panel), in which the mean activity traces and the corresponding errors of AB and TLAB do not overlap, while the errors of the other two comparisons overlap. For the 30-s period after Light-On stimulus (1–30 s; Table 1A), the activity of every strain was different from the others (p-values of all pairwise comparison were < 0.0001). This observation is also supported by the difference of the mean activity traces after reaching their peak value (Fig 2, left panel). For the 30-s period before the Light-Off stimulus (-29–0 s; Table 1A), there was no statistical difference in the activity between AB & TL (p = 0.2048), while there was a difference in the activity between TL & TLAB (p < 0.0001), and AB & TLAB (p = 0.0030). These are corroborated by the corresponding activity plots (Fig 2, right panel), in which the activity of TLAB before light change is noticeably lower than that of AB and TL, while the activity traces of the latter two WT strains highly overlap with each other. For the 30-s period after the Light-Off stimulus (1–30 s; Table 1A), there was a statistical difference in the activity in all three pairwise comparisons of the WT strains (p < 0.0001). As evidenced by the activity plots in Fig 2, TLAB is obviously different from the other two strains because it has a lower sustained activity from 4 to 30 s. While this period of sustained activity is similar between AB and TL, AB has a higher peak activity right after the stimulus change. Thus, the Hotelling’s T-squared test effectively detects activity differences between different strains.

**Table 1:** The Hotelling’s T-squared test of VMR between different strains at 6 dpf.

**A. The Hotelling’s T-squared test of VMR between different strains at 6 dpf**				
Comparison	Test statistic (p-value)			
	Light-On (-29–0 s)	Light-On (1–30 s)	Light-Off (-29–0 s)	Light-Off (1–30 s)
AB VS. TL	1.4083 (0.1017)	**5.1583 (< 0.0001)**	1.1869 (0.2048)	**2.9814 (< 0.0001)**
TL VS. TLAB	1.1478 (0.2572)	**3.5584 (< 0.0001)**	**2.3958 (< 0.0001)**	**2.7308 (< 0.0001)**
AB VS. TLAB	**3.2687 (< 0.0001)**	**2.7563 (< 0.0001)**	**1.9076 (0.0030)**	**4.6229 (< 0.0001)**
**B. The Hotelling’s T-squared test of Light-Off VMR between different strains at 6 dpf using data from two intervals: 1–2 s and 3–30 s**				
	**Light-Off** (1–2 s)		**Light-Off** (3–30 s)	
AB VS. TL	**30.6810 (<0.0001)**		0.9048 (0.6014)	
TL VS. TLAB	0.4844 (0.6116)		**2.5142 (<0.0001)**	
AB VS. TLAB	**21.668 (<0.0001)**		**4.1139 (<0.0001)**	
**C. The Hotelling’s T-squared test of Light-On VMR between different strains at 6 dpf using data only from the first technical repeat**				
	**Light-On** (-29–0 s)		**Light-On** (1–30 s)	
AB VS. TL	**1.9571 (0.0018)**		**5.1843 (< 0.0001)**	
TL VS. TLAB	0.7555 (0.7778)		**2.3645 (< 0.0001)**	
AB VS. TLAB	**1.46 (0.0489)**		**3.7021 (< 0.0001)**	
**D. The Hotelling’s T-squared test of Light-On VMR between different strains at 6 dpf using data from the second and third technical repeats**				
	Light-On (-29–0 s)	Light-On (1–30 s)	Light-Off (-29–0 s)	Light-Off (1–30 s)
AB VS. TL	0.7746 (0.7662)	**2.547 (<0.0001)**	**1.6558 (0.0166)**	**1.8093 (0.0036)**
TL VS. TLAB	**1.9676 (0.0006)**	**2.3505 (<0.0001)**	**2.0502 (0.0006)**	**2.4196 (< 0.0001)**
AB VS. TLAB	**3.0192 (<0.0001)**	**1.4928 (0.0158)**	**1.6301 (0.0166)**	**3.9378 (< 0.0001)**

Even for a short time interval in 30 s, the larval activity can be driven by different neural circuitries. This requires a versatile statistical test that can readily analyze different periods of time. For example, in the first two seconds after light offset, zebrafish larvae display a unique movement termed O-bend, in which the larvae twisted the body to form a circular shape [26]. This O-bend is abolished after eye enucleation, suggesting that this locomotor response is initiated by retina [19]. Unlike this early response, the later sustained response is likely contributed by activity from both retina and extra-ocular photoreceptors [5,19]. Hence, it would be informative to analyze these time frames for the Light-Off stimulus separately. To this end, the Light-Off data were further segregated into 1–2 s and 3–30 s for Hotelling’s T-squared test (Table 1B). The results revealed unique activity differences between different strains during different time periods: during the early 1–2 s, AB’s activity significantly differs from TL and TLAB, while the activity of these latter two strains was similar; whereas during the latter 3–30 s, TLAB’s activity became significantly different from AB and TL, while the activity between the latter two strains was not different from each other. These observations suggest that the O-bend circuitry in AB was slightly different from the other two strains, and that the TLAB might carry unique variation in the locomotor circuitry that gave rise to a different sustained response.

#### 3.1.2. Difference between the same strains across two different time intervals

In addition to comparing two conditions at the same time period, the Hotelling’s T-squared test can also be used to compare two different time periods of the same condition. The utility of this idea is illustrated by the remaining three examples.

***3*.*1*.*2*.*1*. *Example 2*: *The effect of light onset and offset on larval activity***: The activity of the WT larvae was substantially changed by the Light-On and Light-Off stimuli (Fig 2). This can be quantitatively evaluated by the Hotelling’s T-squared test (Table 2A). Specifically, we compared the 30-s activity period before light change with the period after light change. The results reveal that the behavior in each WT strain was significantly different after the Light-On or Light-Off stimulus (p < 0.0001 for all strains). Again, this confirms that each strain displayed a drastic movement in response to the light change (Fig 2).

**Table 2:** The Hotelling’s T-squared test of VMR of the same strain at 6 dpf.

**A. The Hotelling’s T-squared test of VMR of the same strain at 6 dpf before and after light change**			
Comparison	Test statistic (p-value)		
	Strain: AB	Strain: TL	Strain: TLAB
Light-On stimulus (Before light change VS. After light change)	**15.845 (< 0.0001)**	**8.3863 (< 0.0001)**	**14.595 (< 0.0001)**
Light-Off stimulus (Before light change VS. After light change)	**26.881 (< 0.0001)**	**11.784 (< 0.0001)**	**14.984 (< 0.0001)**
**B. The Hotelling’s T-squared test of VMR of the same strain at 6 dpf before and after light change using data only from the first technical repeat**			
	Strain: AB	Strain: TL	Strain: TLAB
Light-On stimulus (Before light change VS. After light change)	**2.8817 (< 0.0001)**	**3.3889 (< 0.0001)**	**5.6767 (< 0.0001)**
**C. The Hotelling’s T-squared test of VMR of the same strain at 6 dpf before and after light change using data from the second and third technical repeats**			
	Strain: AB	Strain: TL	Strain: TLAB
Light-On stimulus (Before light change VS. After light change)	**16.534 (< 0.0001)**	**6.8506 (< 0.0001)**	**11.255 (< 0.0001)**

**Fig 3:**
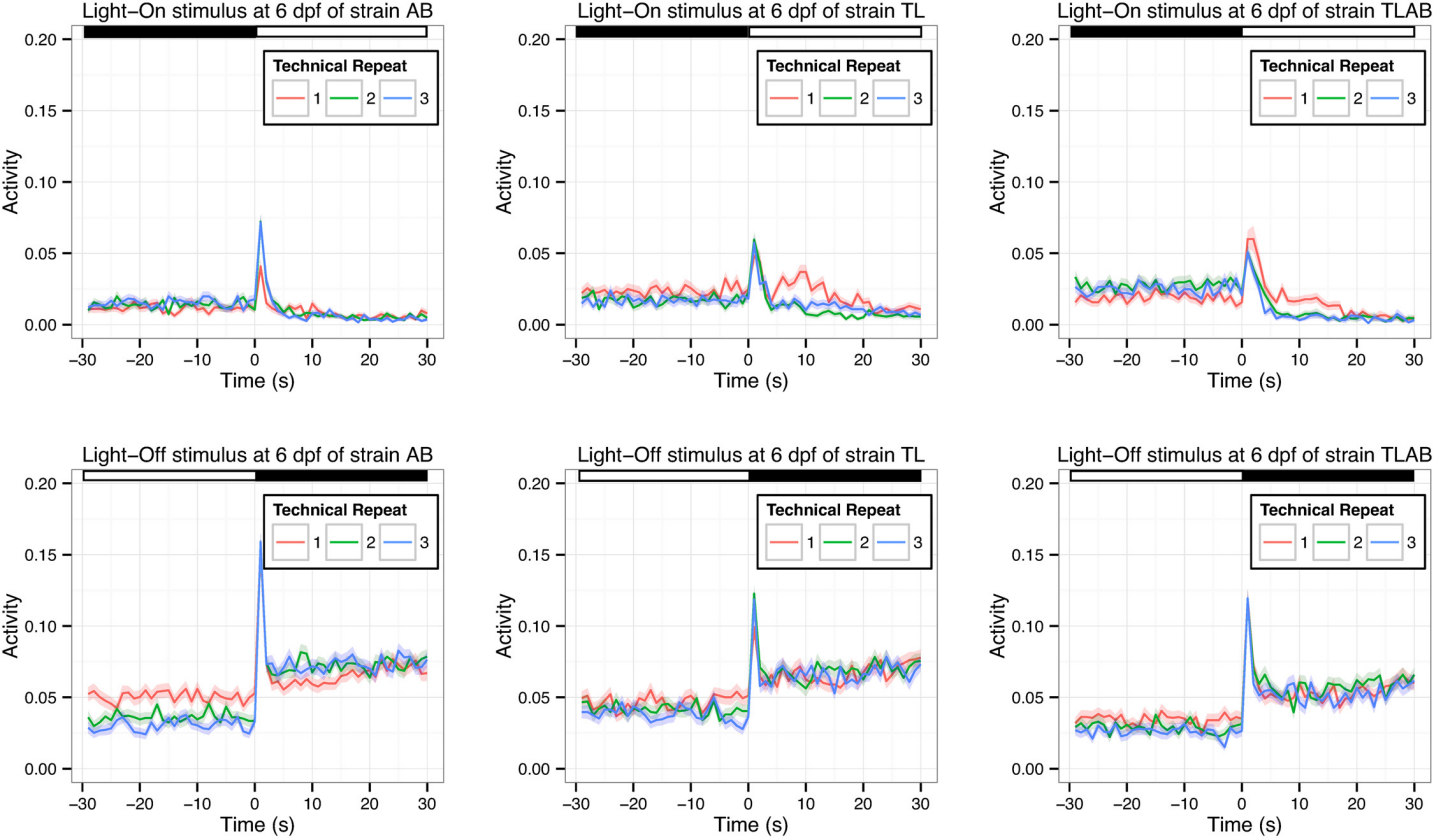
The VMR of 6-dpf WT strains across three sequential technical repeats. The VMR of three WT strains: AB (left), TL (middle) and TLAB (right) was measured as described in the methods. The activity was defined as the fraction of frames in each second that a larva in the video was detected moving (see methods for the detailed definition). There were three sequential technical repeats in the VMR run. For each strain, the activities in each technical repeat were averaged and shown in different colours (repeat 1 = red; repeat 2 = green; repeat 3 = blue). The Light-On and Light-Off results are separately plotted on the top and bottom rows respectively. These plots show the mean activity from 30 s before the light change to 30 s after the light change. The corresponding error in 1 S.D. is shown by the colour ribbon surrounding the mean activity trace. The light and dark periods are indicated by white and black bars at the top of the figures. The sample size was 191 for AB, 176 for TL, and 185 for TLAB respectively. These values are the total observation from the two biological repeats.

***3*.*1*.*2*.*2*. *Example 3*: *Will zebrafish larvae adapt to the light stimulus in multiple technical repeats*?**: Emran and colleagues established the VMR scheme with three sequential technical repeats of the Light-On and Light-Off trials (12)(Fig 1). While the resulting averaged activities from these technical repeats reveal visual defects of mutants (5,14), it is not clear if the larvae would respond identically in each technical repeat. In other words, they may adapt to the light stimuli and display a diminished response upon repeated trials. To investigate this possibility, we analyzed the individual technical repeat of the Light-On VMR (Fig 3, top row) and Light-Off VMR (Fig 3, bottom row) of each strain at 6 dpf, when all strains showed a robust response. Specifically, we compared the 30-s activity periods before and after light change across the three technical replicates (Tables 3 and 4).

**Table 3:** The Hotelling’s T-squared test of Light-On VMR between different technical repeats of the same strain at 6 dpf.

Comparison	Test statistic (p-value)					
	AB (-29–0 s)	AB (1–30 s)	TL (-29–0 s)	TL (1–30 s)	TLAB (-29–0 s)	TLAB (1–30 s)
1^st^ and 2^nd^ technical repeats	1.1676 (0.2556)	**1.6063 (0.0081)**	1.2515 (0.4020)	**3.8094 (<0.0001)**	1.1319 (0.4818)	**1.9414 (0.0015)**
2^nd^ and 3^rd^ technical repeats	1.0737 (0.2556)	1.0745 (0.3678)	0.8081 (0.6930)	1.1042 (0.1498)	0.7348 (0.8132)	0.7925 (0.8136)
1^st^ and 3^rd^ technical repeats	1.2201 (0.2556)	**2.2656 (0.0006)**	1.0895 (0.4476)	**2.5068 (<0.0001)**	1.0711 (0.4818)	**2.8016 (<0.0001)**

**Table 4:** The Hotelling’s T-squared test of Light-Off VMR between different technical repeats of the same strain at 6 dpf.

Comparison	Test statistic (p-value)					
	AB (-29–0 s)	AB (1–30 s)	TL (-29–0 s)	TL (1–30 s)	TLAB (-29–0 s)	TLAB (1–30 s)
1^st^ and 2^nd^ technical repeats	**1.6285 (0.0165)**	1.1920 (0.4932)	0.9494 (0.6322)	1.3927 (0.0735)	1.1059 (0.4206)	1.0339 (0.3870)
2^nd^ and 3^rd^ technical repeats	1.0668 (0.3248)	0.7707 (0.7390)	0.8449 (0.6322)	0.8527 (0.6192)	0.6325 (0.9174)	1.5832 (0.0522)
1^st^ and 3^rd^ technical repeats	**2.5660 (<0.0001)**	0.9971 (0.5688)	1.4448 (0.0984)	1.4203 (0.0735)	1.0969 (0.4206)	1.016 (0.3870)

Table 3 shows the comparisons between these three technical repeats of the Light-On VMR. There was a difference in activity in all three strains after light onset between the 1^st^ and 2^nd^ repeats, and the 1^st^ and 3^rd^ repeats (p < 0.05). There was no difference between the 2^nd^ and 3^rd^ repeats after light onset, or between all repeats before light onset. Thus, the results indicate that the 1^st^ technical repeat of the Light-On VMR was different from the 2^nd^ and 3^rd^ repeats in each strain. Since the 1^st^ technical repeat of the Light-On VMR was statistically different from the other two repeats, we re-analyzed the comparisons of Light-On VMR between different strains, and the comparisons of the same strain before and after light change as originally presented on Tables 1A and 2A respectively. Specifically, the 1^st^ technical repeat was separately analyzed from the other two repeats (Tables 1C, 1D, 2B and 2C). Consistent with the results from all repeats, different strains displayed significantly different activity after light change in the analysis, regardless of using either the 1^st^ technical repeat (Table 1C) or 2^nd^ & 3^rd^ technical repeats (Table 1D). The same situation was observed in the comparisons of the same strain before and after light change (Table 2B and 2C). For the Light-Off VMR, there was no significant difference in activity between the technical repeats of all strains after light offset (Table 4; p > 0.05). Among all comparisons before light offset, only the 1^st^ repeat of AB was different from the 2^nd^ and 3^rd^ repeats (p < 0.05). Thus, these observations suggest that all technical repeats of the Light-Off VMR are comparable in each strain and there is no sign of adaptation.

**Fig 4:**
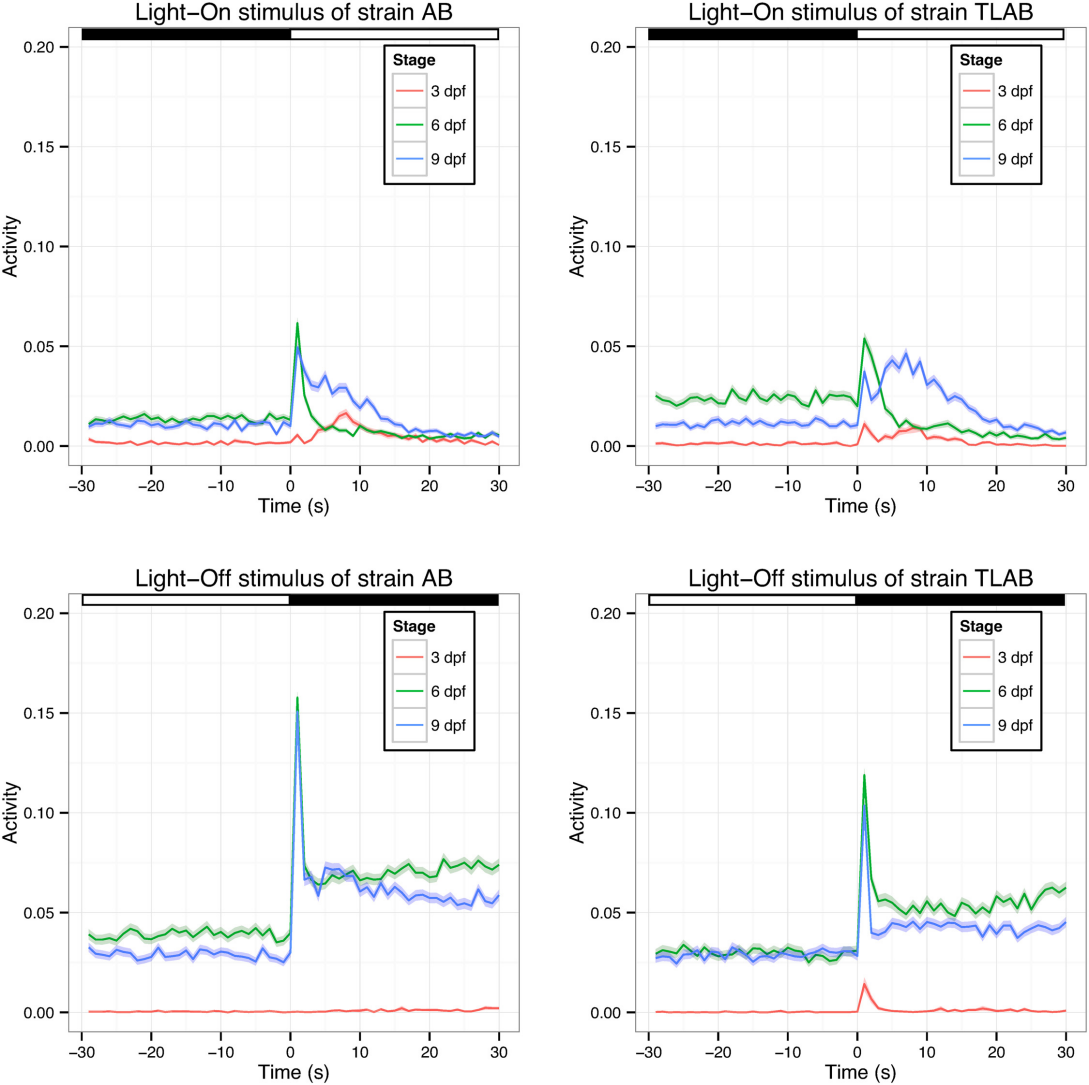
The VMR of AB and TLAB at 3, 6 and 9 dpf. The VMR of AB (left) and TLAB (right) was measured as described in the methods. The activity was defined as the fraction of frames in each second that a larva in the video was detected moving (see methods for the detailed definition). Then, the activities were averaged across all trials under the same light stimulus. The results for Light-On and Light-Off VMR are plotted at the top and bottom row respectively. These figures show the mean activity of 3 dpf (red trace), 6 dpf (green trace) and 9 dpf (blue trace) from 30 s before the light change to 30 s after the light change. The corresponding error in 1 S.D. is shown by the colour ribbon surrounding the mean activity trace. The light and dark periods are indicated by white and black bars at the top of the figures. The sample size was 576 for AB, 576 for TL, and 564 for TLAB respectively at 3 dpf; 573 for AB, 528 for TL, and 555 for TLAB respectively for 6 dpf; and 558 for AB, 519 for TL, and 552 for TLAB respectively for 9 dpf. These values are the total observation from the two biological repeats and three technical repeats.

***3*.*1*.*2*.*3*. *Example 4*: *Difference between different stages of the same strain***: The VMR data were collected from three WT strains each day from 3 to 9 dpf. During this period, the larvae underwent substantial physical development, including locomotor circuit and visual system. This might affect the larval VMR. To determine this difference, we compared the activity of AB and TLAB strains between 3, 6 and 9 dpf (Fig 4). Again, we specifically analyzed the 30-s activity period before and after light change. The results of the Light-On VMR are shown in Table 5A and the top figures of Fig 4. In AB, the activity of each stage was significantly different from the other stages after light change (p < 0.0001). Before light change, the activity of larvae at 6 and 9 dpf was more similar than that between the other stages, but still significantly different (p < 0.05). In TLAB, the activity was significantly different between all stages both before and after light change (p < 0.0001). Furthermore, we separately analyzed the 1^st^ technical repeat and the other two repeats for the Light-On VMR (Table 5B and 5C), as we showed the 1^st^ technical repeat was different from the others (Table 4). In these analyses, all conclusions remained the same, except for the activity of AB at 6 and 9 dpf before light change in the 2^nd^ and 3^rd^ technical repeats. Thus, Light-On stimulus consistently and uniquely induce different locomotor behaviours in different strains at different stages. The corresponding comparisons of the Light-Off VMR are shown in Table 6 and the bottom figures of Fig 4. Here we see that activity of AB before and after the light change was significantly different across different stages (p < 0.0001). The behaviour of TLAB at different stages was significantly different after light change (p < 0.0001). However, before light change, there was a difference in activity between 3 dpf and the other two stages (p < 0.0001), but not between 6 and 9 dpf (p > 0.05).

**Table 5:** The Hotelling’s T-squared test of Light-On VMR between different stages of the same strain.

**A. The Hotelling’s T-squared test of Light-On VMR between different stages of the same strain**				
Comparison	Test statistic (p-value)			
	AB (-29–0 s)	AB (1–30 s)	TLAB (-29–0 s)	TLAB (1–30 s)
3 dpf VS. 6 dpf	**17.851 (< 0.0001)**	**13.494 (< 0.0001)**	**13.617 (< 0.0001)**	**12.944 (< 0.0001)**
6 dpf VS. 9 dpf	**1.6319 (0.0124)**	**12.293 (< 0.0001)**	**4.1654 (< 0.0001)**	**16.358 (< 0.0001)**
3 dpf VS. 9 dpf	**13.147 (< 0.0001)**	**17.398 (< 0.0001)**	**11.594 (< 0.0001)**	**35.358 (< 0.0001)**
**B. The Hotelling’s T-squared test of Light-On VMR between different stages of the same strain using data only from the first technical repeat**				
	AB (-29–0 s)	AB (1–30 s)	TLAB (-29–0 s)	TLAB (1–30 s)
3 dpf VS. 6 dpf	**5.9776 (< 0.0001)**	**4.1605 (< 0.0001)**	**5.0116 (< 0.0001)**	**7.4399 (< 0.0001)**
6 dpf VS. 9 dpf	**1.406 (0.0396)**	**6.1705 (< 0.0001)**	**1.7169 (0.0046)**	**7.2368 (< 0.0001)**
3 dpf VS. 9 dpf	**6.7456 (< 0.0001)**	**5.9348 (< 0.0001)**	**4.4079 (< 0.0001)**	**16.002 (< 0.0001)**
**C. The Hotelling’s T-squared test of Light-On VMR between different stages of the same strain using data from the second and third technical repeats**				
	AB (-29–0 s)	AB (1–30 s)	TLAB (-29–0 s)	TLAB (1–30 s)
3 dpf VS. 6 dpf	**15.208 (< 0.0001)**	**11.384 (< 0.0001)**	**10.943 (< 0.0001)**	**9.1793 (< 0.0001)**
6 dpf VS. 9 dpf	1.3349 (0.073)	**8.1946 (< 0.0001)**	**3.5779 (< 0.0001)**	**13.047 (< 0.0001)**
3 dpf VS. 9 dpf	**9.7053 (< 0.0001)**	**15.538 (< 0.0001)**	**10.421 (< 0.0001)**	**25.398 (< 0.0001)**

**Table 6:** The Hotelling’s T-squared test of Light-Off VMR between different stages of the same strain.

Comparison	Test statistic (p-value)			
	AB (-29–0 s)	AB (1–30 s)	TLAB (-29–0 s)	TLAB (1–30 s)
3 dpf VS. 6 dpf	**37.526 (< 0.0001)**	**151.29 (< 0.0001)**	**16.95 (< 0.0001)**	**43.484 (< 0.0001)**
6 dpf VS. 9 dpf	**2.4207 (< 0.0001)**	**5.4642 (< 0.0001)**	0.96459 (0.5022)	**4.1313 (< 0.0001)**
3 dpf VS. 9 dpf	**42.012 (< 0.0001)**	**168.68 (< 0.0001)**	**29.78 (< 0.0001)**	**51.29 (< 0.0001)**

#### 3.1.3. Power analysis

**Fig 5:**
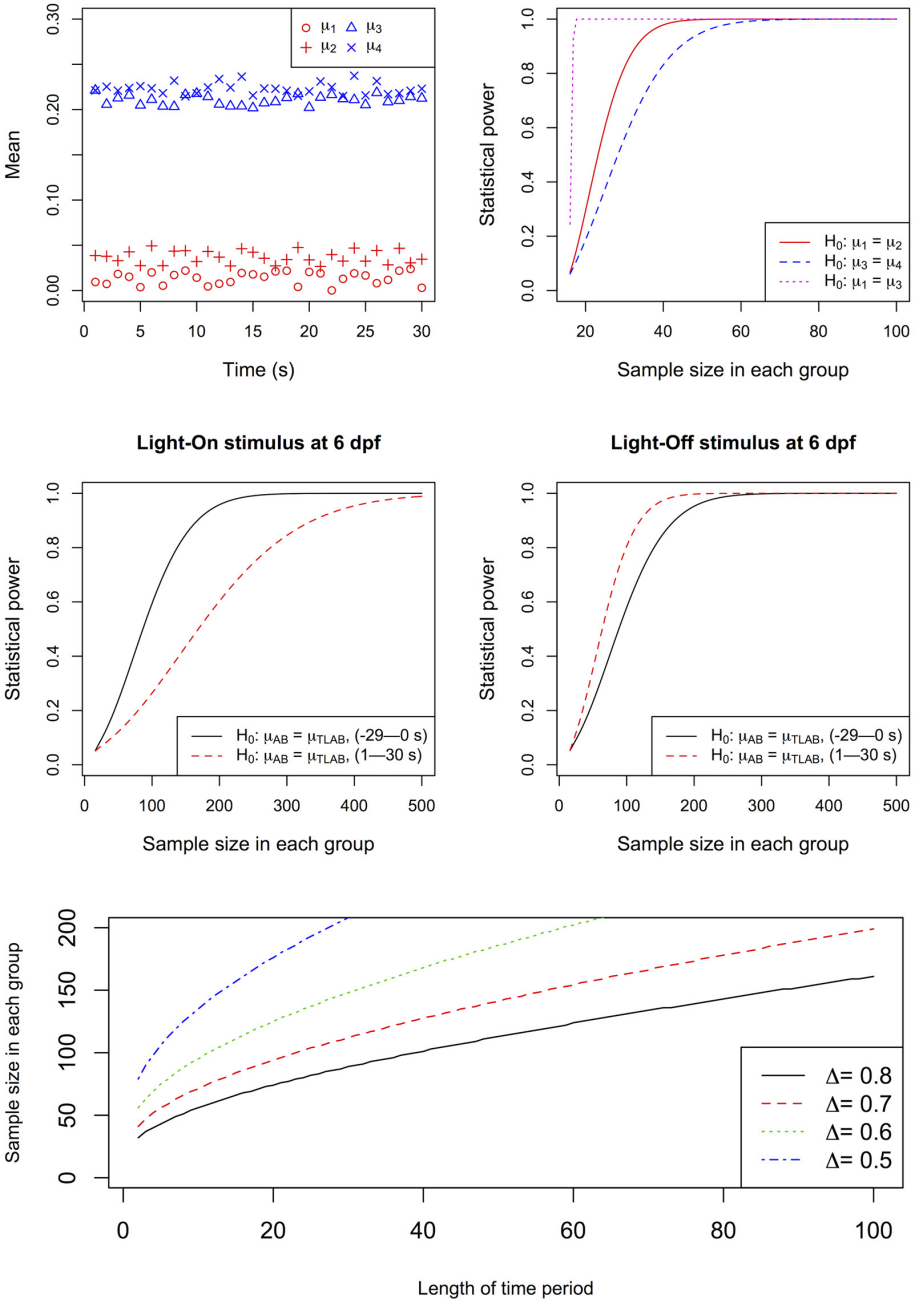
Power analysis. (Top left) Four hypothetical activity profiles. *μ*_1_ (red circles) and *μ*_2_ (red pluses) are two vectors with very small difference and without any overlap. *μ*_3_ (blue triangles) and *μ*_4_ (blue crosses) are two vectors with very small difference and overlap. (Top right) These vectors were used for power analysis of three comparisons: (1) *μ*_1_ vs. *μ*_2_ (red curve); (2) *μ*_3_ vs. *μ*_4_ (blue dash curve); and (3) *μ*_1_ vs. *μ*_3_ (pink dotted curve). In the plot, the y-axis shows the statistical power, while the x-axis shows the sample size in each group. (Middle left) The power analysis results of two comparisons: (1) *μ*_*AB*_ vs. *μ*_*TLAB*_ before light change (-29–0 s) of the Light-On stimulus at 6 dpf (black curve); (2) *μ*_*AB*_ vs. *μ*_*TLAB*_ after light change (1–30 s) of the Light-On stimulus (red dash curve). (Middle right) The power analysis results of two comparisons: (1) *μ*_*AB*_ vs. *μ*_*TLAB*_ before light change of the Light-Off stimulus (black curve); (2) *μ*_*AB*_ vs. *μ*_*TLAB*_ after light change of the Light-Off stimulus (red dash curve). In these four comparisons, the y-axis shows the statistical power, while the x-axis shows the sample size in each group. This sample size can be further reduced in real experiments because they often have multiple biological and technical repeats, which can be combined as indicated by our analyses. For example, one sixth of the samples can be used to attain the same theoretical power if an experiment is conducted with 2 biological repeats and 3 technical repeats, a typical VMR design that was used in this study. These sample numbers are shown in the parenthesis in the x-axis. (Bottom) The relationship between the length of time period and sample size under different effect size. In this simulation, the significance level and statistical power is fixed at 0.05 and 0.8 respectively, and the effect size is calculated as *Δ* = (*μ* ^(1)^ − *μ* ^(2)^)′*Σ* ^−1^(*μ* ^(1)^ − *μ* ^(2)^). Four sample effect sizes (0.5, 0.6, 0.7 and 0.8) were used in the simulation for time period ranges from 2 to 100. The results indicate that as the length of time period becomes shorter, fewer samples are needed to attain statistical significance. The sample size can be further proportionally reduced by proper experimental replications.

To illustrate the performance of Hotelling’s T-squared test, we performed a simple simulation to determine difference between activity profiles. In the simulation, the null hypothesis was *H*_0_:*μ* ^(1)^ = *μ* ^(2)^, where *μ* ^(1)^ and *μ* ^(2)^ were two vectors of activity profile with length t (t is the length of time interval, which is chosen as 30 s in the simulation). Each element in the vectors corresponds to the mean activity at a specific time point along the time interval. Four vectors of length 30 were generated in uniform distribution *U*(*a*, *b*) with *a* and *b* as the minimum and maximum values respectively (Fig 5, top row, left panel). Different values were chosen for *a* and *b* to simulate the following three scenarios for the power analysis of the Hotelling’s T-squared test (Fig 5, top row, right panel):

- ***Scenario 1*: *A very small difference between two vectors without any overlap***. In this simulation, *μ*_1_ ~ *U*(0.1, 0.1250); *μ*_2_ ~ *U*(0.1251, 0.15). These vectors slightly differed from each other and did not overlap. A statistical power of 0.8 could be attained with less than 40 samples.
- ***Scenario 2*: *A very small difference between two vectors with overlap***. In this simulation, *μ*_3_ ~ *U*(0.2, 0.225); *μ*_4_ ~ *U*(0.2125, 0.2375). These vectors had a smaller difference between each other than that between *μ*_1_ and *μ*_2_. Unlike *μ*_1_ and *μ*_2_, *μ*_3_ and *μ*_4_ slightly overlapped with each other. A statistical power of 0.8 could be attained with less than 50 samples.
- ***Scenario 3*: *A large difference between two vectors without any overlap***. The same *μ*_1_ and *μ*_3_ vectors were used in this simulation. They substantially differed from each other and did not overlap. A statistical power of 0.8 could be attained with just 18 samples.
- ***Scenario 4*: *A very small difference between two vectors from real data***. The activity values of AB and TLAB at 6 dpf (Fig 2) were used in this power analysis. The analysis separately evaluated the statistical power needed to declare a difference between AB and TLAB under the Light-On stimulus (Fig 5, middle row, left panel) and Light-Off stimulus (Fig 5, middle row, right panel). For each stimulus, two time periods were used: -29–0 s (before light change), and 1–30 s (after light change). In each time period, the mean activity vectors of AB and TLAB were denoted as *μ*_*AB*_ and *μ*_*TLAB*_ respectively. For Light-On stimulus, a statistical power of 0.8 could be attained with 138 and 276 samples for the time periods -29–0 s and 1–30 s respectively (Fig 5, middle row, left panel). For Light-Off stimulus, a statistical power of 0.8 could be attained with 141 and 100 samples for the time periods -29–0 s and 1–30 s respectively. In our experiments, the dataset was obtained from 2 biological repeats and 3 technical repeats. As discussed in section 3.1.2.2 and section 3.2, these repeats could potentially be combined to increase statistical power. In other words, the number of actual samples could be reduced by replicating the experiments. For instance, in the aforementioned power analysis of AB and TLAB at 6 dpf, the actual number of animals would become 23 (138/6) and 46 (276/6) for Light-On stimulus, and approximately 24 (141/6) and 17 (100/6) for Light-Off stimulus. It should be noted that using data from technical repeats may suffer from pseudoreplication, a scenario that the repeats are not independently measured. This would increase type I error rate and reduce confidence intervals. Even though these issues may be tolerable for a first-pass screening, it is essential to use an appropriate number of independent larvae for critical observations to attain the desirable statistical power.

To further aid selection of sample size for replicated experiments, we plotted the relationship between effect size, length of time period used in analysis, and sample size in each experimental group (Fig 5, bottom row). In this plot, the statistical power was set at 0.8. The results show that fewer samples are needed to gain statistical power of 0.8 as the length of time periods becomes shorter. For example, when the time period is as short as 2 seconds, it only requires 32, 41, 56 and 79 samples in each group given that the effect size equals to 0.8, 0.7, 0.6 and 0.5 respectively. The actual number of animals can be proportionally reduced with appropriate experimental replications. Thus, these power analyses suggest that a relatively small sample size could detect subtle differences between two vectors. As a result, Hotelling’s T-squared test can be an effective tool to analyze similar time-series behavioural data.

### 3.2. MANOVA models

While the Hotelling’s T-squared test provides a statistical measure of the difference between two samples, it does not yield a comprehensive insight into what factors drive those differences. As a complementary analysis, the impact of different experimental factors on activities was analyzed by multi-factor MANOVA models. MANOVA is a multivariate relative of the common ANOVA model, and has been widely used to compare multivariate response in multiple groups [27,28]. Thus, it is particularly suitable for behaviour data like the VMR. Compared to the common ANOVA model, MANOVA model takes into account of the time dependency among response variables and reveals other dependency structures of continuous responses data. Furthermore, it completes the Hotelling’s T-squared test by analyzing all the factors together and identifying the impact of each factor on larval activities.

#### 3.2.1. Effects of multiple factors on larval activity

First, we created a MANOVA model with factors that likely affected larval activity in the VMR assay (Table 7). These factors include Location (well location within the 96-well plate), Biological Repeat, Strain, Stage (dpf), Technical Repeat (total = 3; See Fig 1), Light Stimulus (On vs. Off; See Fig 1), and the synergistic effect among factors of biological variations and factors of treatment effects (i.e. interaction). Some synergistic effects were excluded from the model because they were not deemed biologically meaningful. For example, the location effect should be invariant across different strains, stages, technical repeats and light stimuli. A similar assumption was imposed on the factor Biological Repeat. Thus, the interactions of these two factors with other factors were not included in the model. The data period of the model covers 30 s before light change to 30 s after light change. The results of this MANOVA model are shown in Table 7.

**Table 7:** A MANOVA model of the VMR.

	Pillai*	Pr(>F)
Location	0.3316	**< 0.0001**
Biological Repeat	0.0150	**< 0.0001**
Strain	0.0219	**< 0.0001**
Stage	0.4234	**< 0.0001**
Technical Repeat	0.0173	**< 0.0001**
Light Stimulus	0.3772	**< 0.0001**
Strain:Stage	0.1195	**< 0.0001**
Strain:Technical_Repeat	0.0171	**< 0.0001**
Strain:Light_stimulus	0.0506	**< 0.0001**
Stage:Technical_Repeat	0.0460	**< 0.0001**
Stage:Light_stimulus	0.2452	**< 0.0001**
Technical_Repeat: Light_stimulus	0.0236	**< 0.0001**

*Pillai is the Pillai-Bartlett Trace is the sum of the variance that can be explained by the factors (see Section 2.2.2.3). The sample size in this model is 23268.

The Stage factor had the largest effect size (0.4234; p < 0.0001). As the larvae were growing from 3 to 9 dpf, their locomotor behaviour would steadily mature and differ on different days (Fig 4). This is confirmed by the significant differences in activity between different stages in Tables 5A and 6 (section 3.1.2.3). The Light_Stimulus factor had the second largest effect size (0.3772; p < 0.0001). This factor further distinguished the difference in light change from Off to On (Light-On stimulus), and On to Off (Light-Off stimulus) (Fig 1). The significant effect size indicates that the Light-On and Light-Off stimuli contributed to the locomotor activity differently. The Stage:Light_Stimulus factor had a large and significant effect size (0.2452; p < 0.0001), indicating that differential response to light onset and offset was a function of stage. This is also illustrated in Fig 4 in which 3-dpf larvae obviously did not display much VMR when compared with the later stages. The Location factor also had a large effect size (0.3316; p < 0.0001), suggesting that larvae behaved differently in different wells. Compared with these factors, the other factors had a much smaller effect size. For example, the Biological_Repeat and Technical_Repeat factors had a small effect size (0.0150 and 0.0173; p < 0.0001). Furthermore, the effect size was relatively small in the interaction factors with Technical_Repeat factor (Strain:Technical_Repeat, Stage:Technical_Repeat, & Technical_Repeat:Light_Stimulus; the corresponding effect sizes were 0.0171, 0.046 & 0.0236; p < 0.0001 in all cases). These observations suggest that there were slight variations between independent biological repeats and technical repeats. The Strain factor had a small effect size (0.0219; p < 0.0001), indicating that there were intrinsic differences between different WT strains. This difference was further revealed by the three interaction factors with Strain factor. Two of these factors (Strain:Stage and Strain:Light_Stimulus) had an effect size slightly larger than that of the Strain factor (0.1195 and 0.0506 respectively; p < 0.0001), while the other one (Strain:Technical_Repeat) had a smaller effect size (0.0171; p < 0.0001). Thus, the behaviour of different WT strains differed at different stages, under different light stimuli, and in different technical repeats. This is also indicated by the example activity plots as shown in Fig 4. Together, the model reveals that larval activity was substantially affected by four factors: Stage, Light Stimulus, their interaction, and Location. Among them, only the Stage factor is intrinsic to the animals; the other factors are extrinsic or are a combination of both intrinsic and extrinsic factors.

#### 3.2.2. Effects of multiple factors on Light-On and Light-Off VMR

We are particularly interested in the factors that are related to light stimulus because they allow us to dissect the underlying visual function of the larvae. Hence, we separately analyzed the data for Light-On stimulus (Table 8) and Light-Off stimulus (Table 9). These models tested the effect size of the following factors: Location (well location within the 96-well plate), Biological Repeat, Strain, Stage (dpf), Technical Repeat (1^st^–3^rd^ trials), and the synergistic effect of these factors (i.e. interaction) on larval activity before and after light change. Through this arrangement, we were able to compare effect of these factors on larval activity upon light change.

**Table 8:** A MANOVA model of the Light-On VMR.

	Before light change		After light change	
	Pillai*	Pr(>F)	Pillai*	Pr(>F)
Location	0.2736	**< 0.0001**	0.3184	**< 0.0001**
Biological Repeat	0.0122	**< 0.0001**	0.0172	**< 0.0001**
Strain	0.0134	**< 0.0001**	0.1070	**< 0.0001**
Stage	0.0946	**< 0.0001**	0.3806	**< 0.0001**
Technical Repeat (1^st^-3^rd^)	0.0063	0.1250	0.0523	**< 0.0001**
Strain:Stage	0.0696	**< 0.0001**	0.3042	**< 0.0001**
Strain:Technical Repeat	0.0138	**0.0115**	0.0261	**< 0.0001**
Stage:Technical Repeat	0.0401	**0.0003**	0.0697	**< 0.0001**
Strain:Stage:Technical Repeat	0.0747	**0.0003**	0.0945	**< 0.0001**

*Pillai is the Pillai-Bartlett Trace is the sum of the variance that can be explained by the factors (see Section 2.2.2.3). The sample size in this model is 11634.

**Table 9:** A MANOVA model of the Light-Off VMR.

	Before light change		After light change	
	Pillai*	Pr(>F)	Pillai*	Pr(>F)
Location	0.3194	**< 0.0001**	0.3335	**< 0.0001**
Biological Repeat	0.0013	0.9933	0.0197	**< 0.0001**
Strain	0.0092	**0.0003**	0.0543	**< 0.0001**
Stage	0.3139	**< 0.0001**	0.4821	**< 0.0001**
Technical Repeat	0.0229	**< 0.0001**	0.0063	0.1367
Strain:Stage	0.0511	**< 0.0001**	0.1358	**< 0.0001**
Strain:Technical Repeat	0.0123	0.1105	0.0141	**0.0057**
Stage:Technical Repeat	0.0376	**0.0094**	0.0361	**0.0229**

*Pillai is the Pillai-Bartlett Trace is the sum of the variance that can be explained by the factors (see Section 2.2.2.3). The sample size in this model is 11634.

The results from the Light-On model are summarized in Table 8. First, the Stage factor had the largest effect on larval activity after light change (0.3806, p < 0.0001). However, this factor had a relatively small effect size before light change (0.0946, p < 0.0001). Together, these suggest that there were differences in larval activity towards light onset at different stages. The Location factor had the next largest effect size after light change (0.3184, p < 0.0001), which was not substantially higher than that before light change (0.2736, p < 0.0001). Two factors related to Strain had a large effect size after the light change including Strain (0.1070, p < 0.0001) and Strain:Stage (0.3042, p < 0.0001). Together, these observations indicate that there was an intrinsic difference between different WT strains on light response, and that the difference varied with stage. These outcomes were also corroborated by the difference between strains in the activity plots (Fig 2). The Technical_Repeat factor had a significant impact on larval activity after light change, as the effect size of the factor was significant (0.0523, p < 0.0001). This indicates that the technical repeats did not give identical response. Intriguingly, the Biological_Repeat factor had a smaller effect size (0.0172, p < 0.0001) than the Technical Repeat factor, suggesting that the biological variation between the larvae of the same strain was smaller than the technical variation after light onset. It should be noted that the reverse trend was observed for the Biological_Repeat and Technical_Repeat factors before light change, in which the effect of the former factor was modestly larger than that of the latter (0.0122, p < 0.0001 vs. 0.0063, p < 0.0001). The remaining interaction factors including Strain:Technical_Repeat, Stage:Technical_Repeat, and Strain:Stage:Technical_Repeat all had modest or small effect sizes on larval activity, which were comparable before and after light change.

The results from the Light-Off model are summarized in Table 9. First, similar to the Light-On model, the Stage factor had the largest effect on larval activity after light change (0.4821; p < 0.0001). However, this factor had a comparably large effect size before light change (0.3139; p < 0.0001). Thus, there was not only a stage difference in larval activity upon light offset, but also a stage difference in the light phase before the offset. This is supported by the difference in the baseline activity at the end of the preceding light phase (Fig 2, right; -29–0 s). The Location factor had the next largest effect size, which was fairly constant before (0.3194, p < 0.0001) and after (0.3335, p < 0.0001) light change. This observation suggests that there was a variation in larval activity between wells. The Strain and Strain:Stage factors had the next largest effect sizes (0.0543 & 0.1358, p < 0.0001 for both) after the light change. Thus, there was an intrinsic difference between different WT strains towards light offset, which also varied at different developmental stages (Figs 2 and 4). The Biological_Repeat factor had a small effect (0.0197, p < 0.0001) after light change, suggesting that there was a difference between the Light-Off responses in independent samples. At the same time, the Technical_Repeat factor did not have a significant effect size (p = 0.14) after light change. It should be noted a reverse trend was observed for these factors before light change. For example, the effect sizes of the Biological_Repeat and Technical_Repeat factors were 0.0013 (p = 0.99) and 0.0229 (p < 0.001) respectively. These observations suggest that there was no difference in the response between biological repeats in the last 30 seconds of the preceding light phase, while there was a difference in the response between technical repeats in this period. The remaining interaction factors (Strain:Technical_Repeat and Stage:Technical_Repeat) had small effect sizes on larval activity, which were comparable before and after light change.

Together, the models for Light-On and Light-Off VMR reveal that larval activity during light onset and offset was substantially affected by four factors: Stage, Location, Strain:Stage, and Strain. Except for the Location factor, all other factors are intrinsic property of the larvae.

#### 3.2.3. Dynamic effects of each variable over time

**Fig 6:**
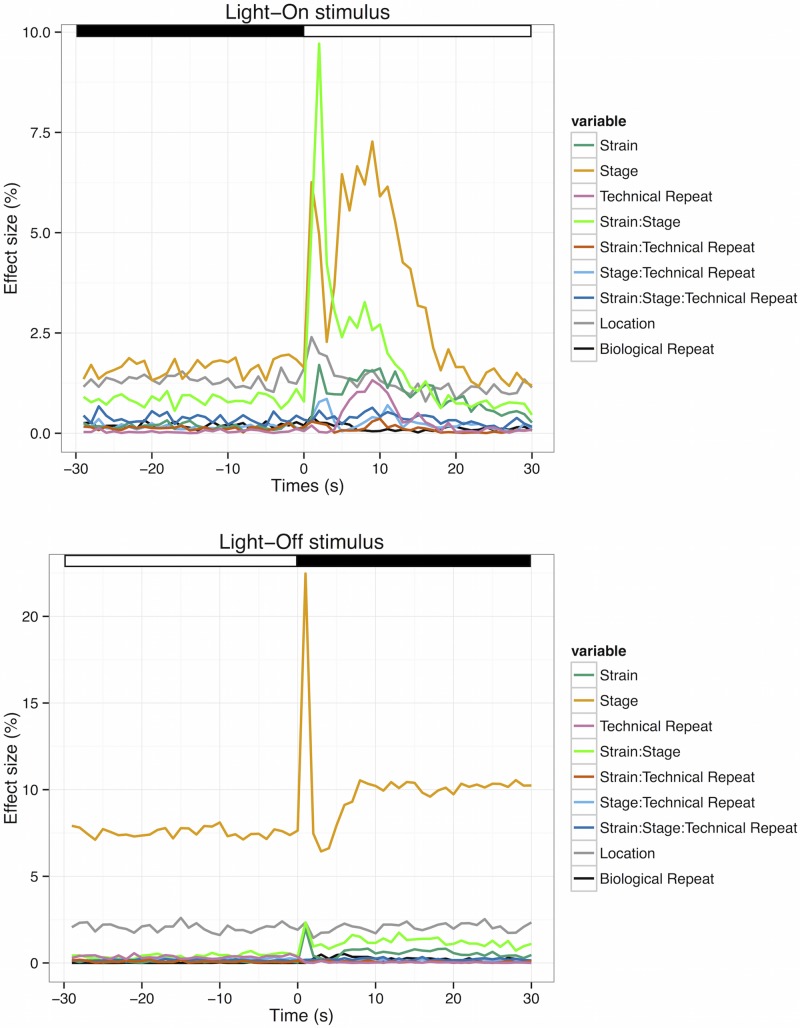
Dynamical effect of different variables on VMR during light change. The dynamic effect size of each factor for Light-On stimulus (top) and Light-Off stimulus (bottom). These figures show the change in effect size of various factors under different light stimuli from 30 s before light change to 30 s after light change. The light and dark periods are indicated by white and black bars at the top of the figures. The sample size in each model is 11634, the same number for the MANOVA models for Light-On and Light-Off VMR (Tables 8 and 9).

While the general MANOVA models give an overview of the effect of different factors on larval activity during a time frame, the effect may vary in each time unit. To study this dynamical change, we used every dependent variable in the MANOVA models to perform a conventional ANOVA at each second from 30 s before light change to 30 s after light change. This analysis reveals the contribution of different variables to the activity over time (Fig 6).

The results of the Light-On stimulus are shown in the top panel of Fig 6. Before light onset, the effect size of each factor was fairly constant. The Stage factor had the largest effect size, which was followed by the Location and Strain:Stage factors. The remaining variables had effect sizes that were nearly zero. After the light onset, the effect sizes of several factors substantially increased. Most strikingly, the Strain:Stage factor contributed to nearly 10% the total variability of activity level in the first 3 s before tapered back down to its previous level at 15 s. The effect size of the Stage factor also increased very rapidly to 6.25% in the first 2 s. Then, it rapidly decreased almost back to the baseline level in the next 2 s, increased to 6.25% again at 5 s, stayed at 6.25%–7.4% for the next 6 s, and gradually returned to the baseline level at 20 s. The Strain factor showed a small increase for about 1% after light onset, which gradually decreased back to the baseline level by 30 s. Both Location and Stage:Technical_Repeat factors had a small 1% increase that lasted until 4 s before returning to the baseline level. The Technical_Repeat factor had a small increase starting from 4 s, reaching a peak level at 1.25%, and returning back to the baseline level at 12.5 s. The Biological_Repeat, Strain:Technical_Repeat and Strain:Stage:Technical_Repeat factors remained fairly constant in their total contributions to the larval activity after the light onset.

The results of the Light-Off stimulus are shown in the bottom panel of Fig 6. The effect sizes of all variables were fairly constant before the light change, just like the situation in the Light-On stimulus. The Stage factor had the largest effect size, at approximately 7.5%. This was followed by the Location factor at approximately 2%–2.5%. The effect sizes of the remaining variables were all close to zero. During light offset, there was an abrupt increase in the effect size of Stage factor to 22.5% in the first 2 s, and an equally as quick return to the original level at 4 s. Then, there was a gradual increase to an elevated level of 10% at 8 s, which was sustained until 30 s. There was also a modest increase in the effect size of the Strain and Strain:Stage factors. The effect size of the Strain factor shared a similar temporal profile as the Stage factor. It peaked at approximately 2% at 2 s, returned to baseline at 4 s, and gradually increased to 1 percent at 6 s, which remained elevated until 30 s. For the Strain:Stage factor, its effect size also peaked at approximately 2.5% at 2 s. Then, it was sustained at around 2% until 30 s. For the remaining factors, there was not a change in their effect size after light offset. In particular, the effect size of the Location factor was still sustained at 2%–2.5%, while the effect sizes for other factors remained close to zero, as before the light change.

## Discussion

The rich repertoire of zebrafish behaviour has been used in many screening studies [2–11] that have generated high-dimensional data. The high dimensionality of the data has created new analytical challenges. These challenges are often originated from the experimental setup, in which the zebrafish larvae are arranged in multi-well plates for simultaneous tracking upon external stimulation. This arrangement is not conducive to standard parametric analyses, including t-tests and ANOVA that are commonly used in the field. At the same time, there are inherent experimental and biological variations in this kind of behavioural data. In this investigation, we have addressed these statistical issues by analyzing the VMR data, a common type of behavioural data, utilizing the Hotelling’s T-squared test. We have also evaluated various factors that may contribute to larval activity by MANOVA.

The Hotelling’s T-squared test compares two time-series data. It confers several advantages in analyzing VMR data: First, it allows for the comparison of multiple time periods within and between treatment conditions (Tables 1–6). Second, the test is sensitive enough to detect a difference between conditions using a small number of larvae (Fig 5), a number that is compatible with commonly-used experimental designs [4,11]. Thus, the test can be readily applied to existing data and new studies that use the typical experimental setup. Third, the test gives a simple significant measure (p-value) for data interpretation. This alleviates the potential difficulty in interpreting complex statistical models. Together, these advantages make the Hotelling’s T-squared test a versatile tool for analyzing VMR data.

The versatility of the Hotelling’s T-squared test was illustrated by several comparisons, using VMR data obtained from different WT strains (Tables 1–6). These comparisons have quantitatively confirmed the qualitative differences observed in the activity plots (Figs 2–4). A few interpretations are summarized here: First, the VMR differed between different WT strains in the first 30 s of light change (Table 1A and Fig 2). Second, the light change induced a significant surge in all WT VMR in the first 30 s (Table 2A and Fig 2). Third, there was no difference between the technical repeats of both Light-On and Light-Off VMR in the first 30 s except for the 1^st^ technical repeat of Light-On VMR (Tables 3 and 4, Fig 3). This 1^st^ technical repeat was different from the other two because it was preceded by a longer 3.5-hr dark adaptation, while the other two repeats were preceded by a shorter 0.5-hr dark phase. In the case of Light-Off VMR, all technical repeats were preceded by a 0.5-hr light phase. Thus, these observations suggest that specific VMR response in a particular trial depends on the length of the preceding stimulus period. Furthermore, technical repeats in the same experiment would not lead to adaptation in larval VMR, provided they have the same preceding stimulus period. Even though the 1^st^ technical repeat of Light-On VMR was statistically different from the other two repeats, their separate analysis led to the same major conclusions as in the analyses using all three repeats (Compare Tables 1A, 2A and 5A with Tables 1C, 1D, 2B, 2C, 5B and 5C). This indicates that all these technical repeats are revealing the same biological differences. Fourth, our power analysis indicates that a small number of samples are needed for the test to attain statistical significance, and that this number is a function of the length of time period used in the analysis, effect size, and the number of experimental repeats (Fig 5). This sample size is congruent with the 96-well plate format and compatible with the numbers commonly used in VMR studies. Fifth, the VMR of the same strain differed between different developmental stages (Tables 5 and 6, Fig 4). This implicates a developmental change in the neural circuitry for controlling locomotor activity and responding to abrupt light change. In fact, zebrafish photoreceptor matures from 3 to 9 dpf. The first detectable startle response appears at 3 dpf [29], while the more complicated optokinetic response becomes robust at 5 dpf [30]. In addition, the development of extraocular photoreceptors during this period will also contribute to the locomotor response [19]. Thus, the older larvae should perceive light change better than the younger ones.

In addition to these direct comparisons, it is also important to assess the impact of factors that would affect activity within a specific time frame. To this end, we developed a MANOVA model for the VMR data that span 30 s before light change to 30 s after light change (Table 7). While all factors affected the activity, four of them were the major ones: Stage, Light Stimulus, their interaction and Location. The Stage and Light_Stimulus factors obviously changed the VMR, as shown by Hotelling’s T-squared test above (Tables 5 and 6, Fig 4 for the Stage factor; Table 2A and Fig 2 for the Light_Stimulus factor). The effect of the Location factor indicates that the experimental setup might cause some variations. In our VMR machine, the 96-well plate was illuminated by an LED panel from below. Even though the panel had a diffuser, the plate might not receive even illumination. This theory was confirmed by measuring the luminance at nine locations on the panel. The brightest locations are in the center of the panel, while the dimmest locations are in the periphery. As a result, the larvae did not receive uniform illumination. To minimize the location impact, larvae should be at least arranged in the 96-well plate such that each experimental group is evenly exposed to areas of higher and lower light intensity. Alternatively, they can be arranged in a checker-box pattern [12]. Nonetheless, this arrangement may not be the most conducive to experiments that have multiple conditions that require constant medium changing. In addition to these major factors, the MANOVA model indicates that the biological and technical repeats had very small and yet significant effect sizes. Nonetheless, the magnitude of their effect size was much smaller than that of the other major factors. Therefore, it is plausible to combine biological replicates obtained on the same machine to increase statistical power. Whereas replicated data obtained from different machines should be first analyzed by a similar MANOVA before combining, or the potential machine effect should be explicitly modeled in the data analysis.

One limitation of the full MANOVA model is that it combines the Light-On and Light-Off stimuli. This combination provides critical insight into the major factors as described above. However, it cannot evaluate how light onset and offset would affect the resulting activity differently. To this end, we further built two MANOVA models for the Light-On and Light-Off stimuli (Tables 8 and 9). As in the full model, these models separately analyze the 30-s time period before and after light change.

In the model for Light-On stimulus (Table 8), the Stage, Strain and their interaction factors accounted for a substantial portion of the activity after light change. This observation suggests that light change played an important role in driving the early Light-On VMR and that different strain and larvae at different stages responded to light onset differently. This validates the observations as reported in several recent behavioural studies on WT zebrafish [9,16,17]. The Location factor had a large effect on activity before light change during the preceding dark phase. Nonetheless, the Location factor had a constant effect before and after light change. Thus, the variation conferred by the Location factor would not affect the correct interpretation of the effect of light change on activity. The effect size of Biological_Repeat factor was the smallest of all tested factors. This supports the interpretation of the full MANOVA model that biological replicates may be combined. The Technical_Repeat factor did not significantly contribute to the activity before the light change. This time period was the last 30 s of the preceding dark stimulus that was at least 30-min long (Fig 1). Thus, the larvae could have acclimatized to the environment just before the light change, regardless of the technical repeats. After light change, the effect size of Technical Repeat factor had a modest increase in magnitude and became significant. This confirms the corresponding analysis by the Hotelling’s T-squared test (Table 3), in which the first technical repeat of the Light-On VMR was different from the other two technical repeats. At the same time, the interaction terms involving the Technical Repeat factor had a similarly small effect size before and after light change. This indicates that the impact of the interaction terms on activity was not related to the light change.

The model for Light-Off stimulus provides some similar and different insights into the effects of various factors on larval activity (Table 9). For example, the Stage, Strain and their interaction factor accounted for a substantial part of the activity after light offset. This again implicates that the light change played an important role in the resulting activity, and that different strains responded to the light offset differently. The effect size of the Location factor was also large in this model. The magnitude of the effect was comparable before and after light change. Thus, this observation confirms the interpretation from the light-ON model, in which there was a variation in larval activity between different wells. Nonetheless, this variation was not related to the strain difference or light change. The effect of the Technical_Repeat factor was not significant after light offset, suggesting that the technical repeats per se were highly consistent and that the Light-Off VMR did not show adaptation over multiple repeats. This is likely due to all the technical repeats were preceded by the light phase with same length (30 min; Fig 1). This interpretation is consistent with the observation made the by Hotelling’s T-squared test, in which all technical repeats for Light-Off VMR were not different from each other (Table 4).

These MANOVA models analyzed the effect of a number of factors immediately before and after the light change. However, the effect was not constant during the course of experiment, as shown by our analysis of the dynamic effect of the variables (Fig 6). The most striking result is that the early Light-On VMR (the first 30 s) was heavily affected by the Stage and Strain:Stage factors, while the early Light-Off VMR was heavily affected by the Stage factor. The effect of the other factors was relatively constant throughout the time period, except for a nominal effect by the Strain factor during Light-On VMR, and Strain:Stage factor on the Light-Off VMR. Thus, the Light-On and Light-Off VMR are driven by intrinsic biological differences, including stage and strain, and are not substantially affected by the random and systematic variations. These properties make the VMR assay, particularly the Light-On response, potentially ideal for studies that rely on rapid responses to study visual behaviours or screen eye drugs [5,11,14]. The Light-Off response, however, should be used with caution now. When it is implemented in the way outlined in this study, it can reveal a substantial eye-level contribution, as an eyeless mutant displayed very minimal response under this particular setting up to approximately 30 s after light offset [5]. However, when the activity was summarized in minutes instead of seconds, the same eyeless mutant displayed substantial locomotor behaviour [19]. Thus, these studies indicate that both extraocular photoreceptors and regular photoreceptors contribute to the locomotor response during light offset. To effectively measure eye-level contribution for Light-Off response, we recommend using the settings we outlined in this study, restricting the analysis to data within the first 30 s, or even just the first 2 s to detect the eye-driven O-bend movement [26], and following up interesting observations with other complementary experiments.

In short, this study has developed a statistical framework that applies several established statistical tools for time-series locomotor analysis in zebrafish: a Hotelling’s T-squared test for two time-series comparison, and a MANOVA model for assessing factors that may affect the locomotor activity. These tests are compatible with the commonly-used experimental design and sample numbers. While the evaluation of the tests was conducted with a VMR dataset, these tests should be applicable to any time-series locomotor analysis with similar data structure. Our analysis has also unveiled a potential effect of the location of larvae in the plate on the resulting larval activity. The effect seems to be constant across the experimental time, and should not affect the interpretation of results obtained from our analysis framework. Nonetheless, future analysis should include of the location effect in the Hotelling’s T-squared test. For example, this can be achieved by scaling the activity levels of zebrafish by their location effect before performing the Hotelling’s T-squared test. We will also explore non-parametric analyses for time-series locomotor data with data structure that may not be fully compatible with the framework established in this study. As of now, the establishment of these parametric analyses will immediately facilitate efficient data analysis in high-throughput behavioural studies, studies that would lead to new insights into neurobiology, pharmacology and toxicology.

## Supporting Information

S1 File The R analysis codes used in this study. (ZIP)

## References

[1] Patton EE, Zon LI. The art and design of genetic screens: zebrafish. Nat Rev Genet. 2001;2: 956–966. 11733748 10.1038/35103567

[2] Prober DA, Rihel J, Onah AA, Sung R-J, Schier AF. Hypocretin/orexin overexpression induces an insomnia-like phenotype in zebrafish. J Neurosci. 2006;26: 13400–13410. 10.1523/JNEUROSCI.4332-06.2006 17182791 PMC6675014

[3] Kokel D, Bryan J, Laggner C, White R, Cheung CYJ, Mateus R, et al Rapid behavior-based identification of neuroactive small molecules in the zebrafish. Nat Chem Biol. 2010;6: 231–237. 10.1038/nchembio.307 20081854 PMC2834185

[4] Rihel J, Prober DA, Arvanites A, Lam K, Zimmerman S, Jang S, et al Zebrafish behavioral profiling links drugs to biological targets and rest/wake regulation. Science. 2010;327: 348–351. 10.1126/science.1183090 20075256 PMC2830481

[5] Emran F, Rihel J, Adolph AR, Wong KY, Kraves S, Dowling JE. OFF ganglion cells cannot drive the optokinetic reflex in zebrafish. Proc Natl Acad Sci U S A. 2007;104: 19126–19131. 10.1073/pnas.0709337104 18025459 PMC2141919

[6] Rihel J, Schier AF. Behavioral screening for neuroactive drugs in zebrafish. Dev Neurobiol. 2012;72: 373–385. 10.1002/dneu.20910 21567979

[7] MacPhail RC, Brooks J, Hunter DL, Padnos B, Irons TD, Padilla S. Locomotion in larval zebrafish: Influence of time of day, lighting and ethanol. Neurotoxicology. 2009;30: 52–58. 10.1016/j.neuro.2008.09.011 18952124

[8] Ali S, Champagne DL, Richardson MK. Behavioral profiling of zebrafish embryos exposed to a panel of 60 water-soluble compounds. Behav Brain Res. 2012;228: 272–283. 10.1016/j.bbr.2011.11.020 22138507

[9] De Esch C, van der Linde H, Slieker R, Willemsen R, Wolterbeek A, Woutersen R, et al Locomotor activity assay in zebrafish larvae: influence of age, strain and ethanol. Neurotoxicol Teratol. 2012;34: 425–433. 10.1016/j.ntt.2012.03.002 22484456

[10] Ali S, van Mil HGJ, Richardson MK. Large-scale assessment of the zebrafish embryo as a possible predictive model in toxicity testing. PLoS One. 2011;6: e21076 10.1371/journal.pone.0021076 21738604 PMC3125172

[11] Deeti S, O’Farrell S, Kennedy BN. Early safety assessment of human oculotoxic drugs using the zebrafish visualmotor response. J Pharmacol Toxicol Methods. 2014;69: 1–8. 10.1016/j.vascn.2013.09.002 24091134

[12] Emran F, Rihel J, Dowling JE. A behavioral assay to measure responsiveness of zebrafish to changes in light intensities. J Vis Exp. 2008; pii:923 10.3791/923 PMC2879884 19078942

[13] Gao Y, Chan RHM, Chow TWS, Zhang L, Bonilla S, Pang C-P, et al A high-throughput zebrafish screening method for visual mutants by light-induced locomotor response. IEEE/ACM Trans Comput Biol Bioinform. 2013; Under review. 10.1109/TCBB.2014.2306829 26356340

[14] Zhang L, Chong L, Cho J, Liao P, Shen F, Leung YF. Drug Screening to Treat Early-Onset Eye Diseases : Can Zebrafish Expedite the Discovery ? Asia-Pac J Ophthalmol. 2012;1: 374–383. 10.1097/APO.0b013e31827a9969 26107731

[15] Maurer CM, Schönthaler HB, Mueller KP, Neuhauss SCF. Distinct retinal deficits in a zebrafish pyruvate dehydrogenase-deficient mutant. J Neurosci. 2010;30: 11962–11972. 10.1523/JNEUROSCI.2848-10.2010 20826660 PMC6633552

[16] Lange M, Neuzeret F, Fabreges B, Froc C, Bedu S, Bally-Cuif L, et al Inter-individual and inter-strain variations in zebrafish locomotor ontogeny. PLoS One. 2013;8: e70172 10.1371/journal.pone.0070172 23950910 PMC3739779

[17] Vignet C, Bégout M-L, Péan S, Lyphout L, Leguay D, Cousin X. Systematic screening of behavioral responses in two zebrafish strains. Zebrafish. 2013;10: 365–375. 10.1089/zeb.2013.0871 23738739

[18] Padilla S, Hunter DL, Padnos B, Frady S, MacPhail RC. Assessing locomotor activity in larval zebrafish: Influence of extrinsic and intrinsic variables. Neurotoxicol Teratol. 2011;33: 624–630. 10.1016/j.ntt.2011.08.005 21871562

[19] Fernandes AM, Fero K, Arrenberg AB, Bergeron SA, Driever W, Burgess HA. Deep Brain Photoreceptors Control Light-Seeking Behavior in Zebrafish Larvae. Curr Biol. 2012;22: 2042–2047. 10.1016/j.cub.2012.08.016 23000151 PMC3494761

[20] Beker van Woudenberg A, Wolterbeek A, Te Brake L, Snel C, Menke A, Rubingh C, et al A category approach to predicting the developmental (neuro) toxicity of organotin compounds: the value of the zebrafish (Danio rerio) embryotoxicity test (ZET). Reprod Toxicol. 2013;41: 35–44. 10.1016/j.reprotox.2013.06.067 23796951

[21] Rihel J, Prober DA, Schier AF. Monitoring sleep and arousal in zebrafish. Methods Cell Biol. 2010;100: 281–294. 10.1016/B978-0-12-384892-5.00011-6 21111222

[22] Zhou Y, Cattley RT, Cario CL, Bai Q, Burton EA. Quantification of larval zebrafish motor function in multiwell plates using open-source MATLAB applications. Nat Protoc. 2014;9: 1533–1548. 10.1038/nprot.2014.094 24901738 PMC4169233

[23] Hensley MR, Leung YF. A convenient dry feed for raising zebrafish larvae. Zebrafish. 2010;7: 219–231. 10.1089/zeb.2010.0652 20441525

[24] Benjamini Y, Hochberg Y. Controlling the False Discovery Rate: A Practical and Powerful Approach to Multiple Testing. J R Stat Soc Ser B. 1995;57: 289–300.

[25] Anderson TW. An introduction to multivariate statistical analysis. New York: Wiley; 1958.

[26] Burgess HA, Granato M. Modulation of locomotor activity in larval zebrafish during light adaptation. J Exp Biol. 2007;210: 2526–2539. 10.1242/jeb.003939 17601957

[27] Scheiner SM. MANOVA: multiple response variables and multispecies interactions. Des Anal Ecol Exp. 1993;94.

[28] O’Brien RG, Kaiser MK. MANOVA method for analyzing repeated measures designs: an extensive primer. Psychol Bull. 1985;97: 316–333. 3983301

[29] Easter SS Jr, Nicola GN. The development of vision in the zebrafish (Danio rerio). Dev Biol. 1996;180: 646–663. 8954734 10.1006/dbio.1996.0335

[30] Easter SS, Nicola GN. The development of eye movements in the zebrafish (Danio rerio). Dev Psychobiol. 1997;31: 267–276. 9413674 10.1002/(sici)1098-2302(199712)31:4<267::aid-dev4>3.0.co;2-p

